# Pre-eclampsia Complicated With Maternal Renal Dysfunction Is Associated With Poor Neurological Development at 3 Years Old in Children Born Before 34 Weeks of Gestation

**DOI:** 10.3389/fped.2021.624323

**Published:** 2021-04-29

**Authors:** Noriko Yoneda, Satoshi Yoneda, Sayaka Tsuda, Mika Ito, Arihiro Shiozaki, Hideki Niimi, Taketoshi Yoshida, Akitoshi Nakashima, Shigeru Saito

**Affiliations:** ^1^Department of Obstetrics and Gynecology, University of Toyama, Toyama, Japan; ^2^Clinical Laboratory Center, Toyama University Hospital, Toyama, Japan; ^3^Division of Neonatology, Maternal and Perinatal Center, Toyama University Hospital, Toyama, Japan; ^4^University of Toyama, Toyama, Japan

**Keywords:** maternal renal dysfunction, neurological development, pre-eclampsia, preterm delivery, small for gestational age

## Abstract

**Objective:** The purpose of this study was to investigate perinatal factors associated with a poor neurodevelopmental outcome in preterm infants.

**Methods:** A retrospective study was conducted by searching our clinical database between January 2006 and December 2016. A total of 165 singleton children who were born between 23 and 33 weeks of gestation were included. We defined poor neurological development outcomes as follows: cerebral palsy; intellectual disability; developmental disorder including autism and attention-deficit/hyperactivity disorder; low score (<85 points) on Bayley Scales of Infant and Toddler Development, Third Edition (Bayley-III); or low score of Kyoto Scale of Psychological Development corrected at 3 years old. We diagnosed maternal renal dysfunction according to the Clinical Practice Guideline for chronic kidney disease 2018 and the Best Practice Guide 2015 for Care and Treatment of Hypertension in Pregnancy.

**Results:** The rate of poor neurological development was 25/165 (15.2%): cerebral palsy (*n* = 1), intellectual disability (*n* = 1), developmental disorder (*n* = 2), low score of Bayley-III (*n* = 20), and low score of Kyoto Scale of Psychological Development (*n* = 1). Preeclampsia complicated with maternal renal dysfunction (*P* = 0.045) and delivery at <30 weeks of gestation (*P* = 0.007) were independent risk factors for poor neurological development.

**Conclusions:** In addition to previous risk factors such as delivery at <30 weeks of gestation, preeclampsia complicated with renal dysfunction was also associated with poor neurodevelopmental outcomes corrected at 3 years old.

## Introduction

Poor neurological development of preterm infants is related to delivery weeks (1, 2), fetal growth restriction (3, 4), chorioamnionitis, maternal infection (5), maternal increased body mass index (BMI) (6), socioeconomic disadvantage (7, 8), not receiving breast milk at discharge (7), neonatal intensive care unit (NICU) noise (9), longer intensive care unit stay, more complex forms of congenital heart disease (8), and maternal preeclampsia (PE) (10, 11).

Children born to mothers with PE show developmental disorders (10, 11) that contain cognitive, behavioral, and mood deficits compared with offspring from mothers with non-complicated pregnancies (12). Severe PE is a disease complicated with hypertension and proteinuria with or without kidney and liver damage, oliguria, PE, cerebral edema, and cerebral hemorrhage. This will lead to perinatal and neonatal problems, including preterm birth, fetal growth restriction (FGR), reduced birth weight, and NICU stays.

There is also the Baker hypothesis that undernutrition during pregnancy increases the risk of adult diseases (13). Barker (13) demonstrated that many human fetuses have to adapt to a limited supply of nutrients, and so they change their metabolism and physiology. These changes may be the origins of many diseases in later life, including diabetes, hypertension, and coronary heart disease (13).

In Japan, the examination of neurological development is usually assessed by the Tsumori-Inage Infant's developmental test (14) or Kyoto Scale of Psychological Development (15). However, these tests are unique to Japan, and so studies cannot be compared internationally.

Bayley Scales of Infant and Toddler Development, Third Edition (Bayley-III), is an independently examined test designed to assess developmental functioning of infants and toddlers. Bayley*-*III checks development in five areas: cognitive, language, motor, social–emotional, and adaptive behavior (16). Bayley-III is globally used as a method for evaluating development from 1 month to the age of 3.5 years, and there are many reports (17–20).

The objective of this study was to examine the association of perinatal factors and neurological developmental outcomes evaluated by Bayley-III in preterm infants. We examined just one case by Kyoto Scale of Psychological Development.

## Materials and Methods

**Figure 1:**
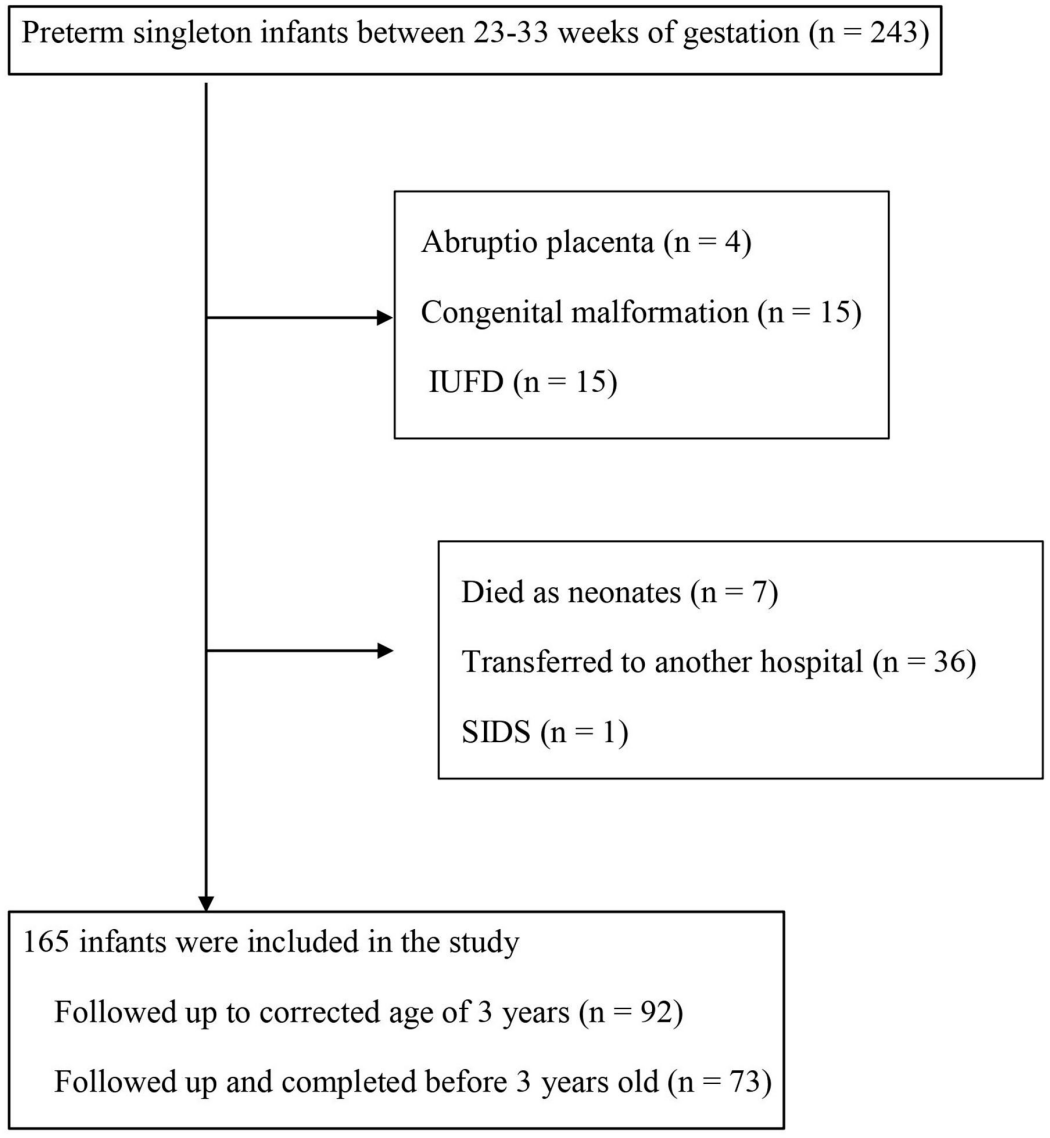
Flow diagram of the study population. IUFD, intrauterine fetal death; SIDS, sudden infant death syndrome.

This was a retrospective study, conducted by examining hospitalized patients in Toyama University Hospital, Department of Obstetrics and Gynecology, between January 2006 and December 2016. Figure 1 shows a flow diagram of the study population. The study group consisted of 243 infants delivered between 23 and 33 weeks of gestation. We excluded cases with children born to mothers with abruptio placenta (*n* = 4), with congenital malformation (*n* = 15), intrauterine fetal death (IUFD) (*n* = 15), who died as neonates (*n* = 7), those transferred to another hospital (*n* = 36), and cases of sudden infant death syndrome (SIDS) (*n* = 1). A total of 165 infants were included in this study; 92 cases were followed up to corrected age of 3 years, and 73 cases were followed up to completion before 3 years old (Figure 1). Informed consent for clinical data, purpose of the study, etc. were signed by all patients. The research protocols were accepted by the institutional review board of Toyama University Hospital.

## Histological Examination of the Placenta

We defined chorioamnionitis according to acute inflammatory cell infiltration in the chorion-decidua and amnion by Blanc's classification: deciduitis (stage I), chorionitis (stage II), and amnionitis (stage III) (21).

### Maternal Factors

PE was diagnosed by the new onset of hypertension and proteinuria or fetal growth restriction or end-organ dysfunction after 20 weeks' gestation. Gestational hypertension (GH) was defined by only hypertension. Hypertension during pregnancy was diagnosed as systolic blood pressure over 140 mmHg and/or diastolic blood pressure over 90 mmHg. Proteinuria was defined by over 300 mg/day. Superimposed PE was diagnosed as chronic hypertension before 20 weeks' gestation and the new onset of proteinuria and/or end-organ dysfunction after 20 weeks' gestation. Preterm premature rupture of membranes (PROM) was diagnosed by the rupture of membranes before 37 weeks' gestation. Preterm PROM was diagnosed to verify the pooling of amniotic fluid in the vagina or the presence of insulin-like growth factor binding protein-1 (IGFBP-1) (Check PROM test; MedixBiochemica, Finland). Clinical chorioamnionitis was diagnosed according to Lencki's criteria (22).

### Neonatal Factors

Respiratory distress syndrome (RDS) was defined by radiographic findings and the administration of surfactant. Chronic lung disease (CLD) was diagnosed by over 28 days' age supplemental oxygen and/or pneumonia; intraventricular hemorrhage (IVH) was diagnosed by cranial ultrasound. Periventricular leukomalacia (PVL) was defined by magnetic resonance imaging and head ultrasound according to injury of deep cerebral white matter and focal necrosis. Necrotizing enterocolitis (NEC) was diagnosed by portal venous gas and pneumatosis. Retinopathy of prematurity (ROP) was identified by retinoscopy, and all patients were treated with laser photocoagulation or anti-vascular endothelial growth factor (VEGF) drugs. Mechanical ventilation was administered for managing preterm neonates with RDS. Non-invasive positive pressure ventilation (NPPV) was used to help remove invasive mechanical ventilation or positive airway pressure and/or inspiratory pressure support. Circulation problem was defined by the need for surgical ligation of patent ductus arteriosus (PDA) and/or late-onset glucocorticoid-responsive circulatory collapse. Postnatal infection was defined by the presence of bacteria in blood culture. Visual impairment and hearing impairment were identified at 3 years old. Visual impairment included amblyopia and squint. Hearing impairment was diagnosed by hearing test.

### Developmental Follow-Up

Neurodevelopmental outcomes were examined by Bayley-III. Composite scores were obtained for motor, cognitive, and emotion domains, with a normal mean of 100 and SD of 15. We defined poor neurodevelopmental outcomes as follows: cerebral palsy, intellectual disability, developmental disorder including autism and attention-deficit/hyperactivity disorder (ADHD), and low score (<85 points) of Bayley-III. One patient was examined by Kyoto Scale of Psychological Development. Kyoto Scale of Psychological Development is one of the most generally used developmental examinations in Japan. This test is an individualized person-to-person test examined by expert psychologists to check a child's development in three areas: Cognitive-Adaptive, Language-Social, and Postural-Motor (15). A total of 73 cases were followed up to completion before 3 years old. Of these 73 cases, all cases were diagnosed as normal development. Clinical examination by the neonatologist and parental questionnaires were inspected at follow-up. Infants who were followed up and completed before 3 years old were instructed by the neonatologists to go and see a neonatologist/pediatrician in our hospital if they had developmental problems at health checkup at 3 years old.

### Diagnosis of Maternal Renal Dysfunction

We diagnosed maternal renal dysfunction as follows: at least one of creatinine clearance under 70 ml/min, oliguria ≤ 400 ml/day, urine protein ≥5 g/day, and pleural effusion and ascites according to the Clinical Practice Guideline for chronic kidney disease (CKD) 2018 (23) and the Best Practice Guide 2015 for Care and Treatment of Hypertension in Pregnancy (24).

### Statistical Analysis

To compare clinical variables between both outcome groups, we used the Fisher's exact test or chi-square test. We used the Mann–Whitney *U* test and Kruskal–Wallis test to compare the differences in continuous variables. We checked the association between perinatal factors and poor neurological outcomes by multiple logistic regression analysis. We adjusted odds ratios (ORs) and 95% confidence intervals (95% CIs). All analyses were operated by statistical analysis software JMP 11.2.0 (SAS Institute Inc., Tokyo, Japan). Significant difference was described as *P* < 0.05.

## Results

**Figure 2:**
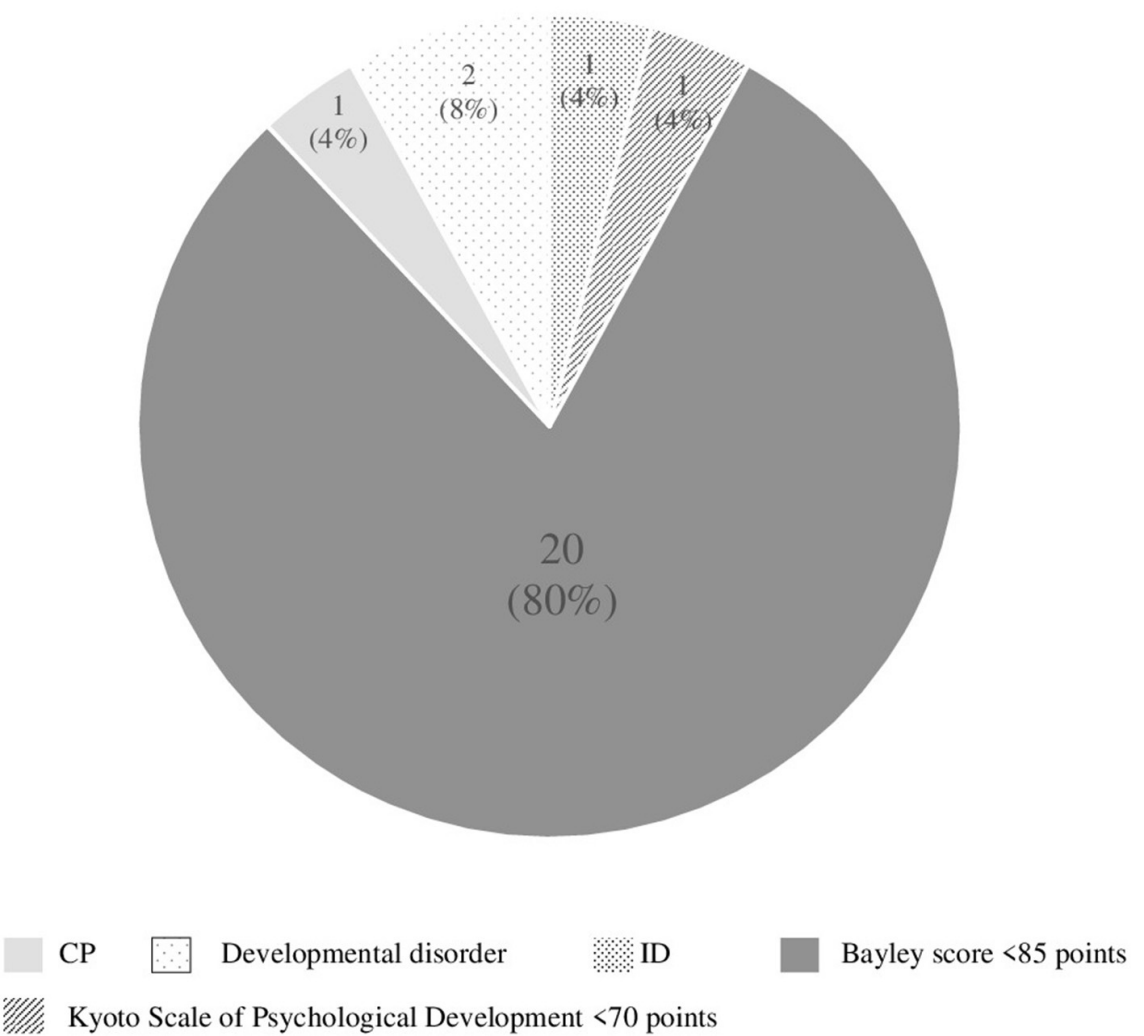
Details of poor neurological development cases (*n* = 25). CP, cerebral palsy; ID, intellectual disability.

Poor neurological development was noted in 25 cases (15.2%) of 165 infants as follows: cerebral palsy (*n* = 1), intellectual disability (*n* = 1), developmental disorder including autism and ADHD (*n* = 2), low score (<85 points) of Bayley-III (*n* = 20), and low score of Kyoto Scale of Psychological Development (*n* = 1) (Figure 2). A total of 91 cases were examined by Bayley-III, and one case was examined by Kyoto Scale of Psychological Development. High grades of developmental delay were: ID (*n* = 1), CP (*n* = 1), developmental disorder (*n* = 2), Bayley-III score <70 points (*n* = 4), and 50 points of Kyoto Scale of Psychological Development (*n* = 1) at a corrected age of 3 years. Mild developmental delay was noted in 16 cases with Bayley-III scores of 70–84 points.

### Risk Factors for Poor Neurological Development in Preterm Infants

Table 1 demonstrates perinatal factors according to neurological development. The delivery week was significantly earlier in the poor outcome group (*n* = 25) than that in the good outcome group (*n* = 140) (28 vs. 30, respectively; *P* = 0.012). No significant differences were observed between the good outcome group and the poor outcome group in clinical characteristics including pre-pregnancy BMI (20.4 vs. 19.9, respectively, *P* = 0.487), pre-delivery BMI (22.8 vs. 22.3, respectively; *P* = 0.505), gestational weight gain (kg) (5.2 vs. 4.8, respectively; *P* = 0.883), rates of gestational diabetes mellitus (%) (2.1 vs. 4.0, respectively; *P* = 0.578), hypothyroidism (%) (2.1 vs. 0, respectively; *P* = 0.460), spontaneous preterm delivery (%) (69.3 vs. 56, respectively; *P* = 0.192), clinical chorioamnionitis (%) (11.4 vs. 12.0, respectively; *P* = 0.934), histological chorioamnionitis (%) (64.5 vs. 56, respectively; *P* = 0.418), funisitis (%) (39.9 vs. 40, respectively; *P* = 0.989), antenatal corticosteroid use (%) (68.6 vs. 68, respectively; *P* = 0.955), and cesarean section (%) (80.7 vs. 80, respectively; *P* = 0.934). The rates of preterm PROM (%) were higher in the good outcome group than those in the poor outcome group (46.4 vs. 24, respectively; *P* = 0.037).

**Table 1:** Perinatal factors according to neurological development.

	**Good (*n* = 140)**	**Poor (*n* = 25)**	***P***
Delivery weeks (weeks)	30 (23–33)	28 (23–32)	**0.012**
Pre-pregnancy BMI	20.4 (13.7–34.4)	19.9 (17.1–25.6)	0.487
Pre-delivery BMI	22.8 (15.6–38.4)	22.3 (18.8–32.7)	0.505
Gestational weight gain (kg)	5.2 (−6–17)	4.8 (−1–17.6)	0.883
Gestational diabetes mellitus (*n*/%)	3 (2.1)	1 (4.0)	0.578
Hypothyroidism (*n*/%)	3 (2.1)	0 (0)	0.460
Early-onset PE, Superimposed PE (*n*/%)	12 (8.6)	5 (20.0)	0.143
GH (*n*/%)	6 (4.3)	2 (8.0)	0.348
PE with maternal renal dysfunction (*n*/%)	3 (2.1)	3 (12.0)	**0.045**
Preterm PROM (*n*/%)	65 (46.4)	6 (24.0)	**0.037**
Spontaneous preterm delivery (*n*/%)	97 (69.3)	14 (56.0)	0.192
Clinical chorioamnionitis (*n*/%)	16 (11.4)	3 (12.0)	0.934
Histological chorioamnionitis (*n*/%)	89/138 (64.5)	14 (56.0)	0.418
Funisitis (*n*/%)	55/138 (39.9)	10 (40.0)	0.989
Antenatal corticosteroid (*n*/%)	96 (68.6)	17 (68.0)	0.955
Cesarean section (*n*/%)	113 (80.7)	20 (80.0)	0.934

*BMI, body mass index; PE, preeclampsia; GH, gestational hypertension; PROM, premature rupture of the membranes. The bold values means P < 0.05*.

The rates of early-onset PE including superimposed PE (%) (8.6 vs. 20, respectively; *P* = 0.143) and GH (%) (4.3 vs. 8, respectively; *P* = 0.348) were not related to poor neurological development. On the other hand, rates of PE with maternal renal dysfunction (%) was significantly higher in the poor outcome group than those in the good outcome group (12 vs. 2.1, respectively; *P* = 0.045). Univariable analyses revealed that preterm infants born early to PE mothers with renal dysfunction were more likely to show poor neurological development (Table 1).

Of the neonatal factors examined, low birth weight (g) (1,017 vs. 1,394, respectively; *P* = 0.001), rates of small for gestational age (SGA) (%) (36 vs. 17.9, respectively; *P* = 0.039), head circumference (HC) at birth (cm) (26.0 vs. 28.0, respectively; *P* = 0.002), rates of PVL (%) (16 vs. 3, respectively; *P* = 0.011), RDS (%) (80.0 vs. 57.1, respectively; *P* = 0.031), NEC (%) (12.0 vs. 1.4, respectively; *P* = 0.005), mechanical ventilation (%) (84.0 vs. 62.9, respectively; *P* = 0.034), duration of oxygen supplementation (days) (71 vs. 35, respectively; *P* = 0.020), and duration of NICU stay (days) (92 vs. 67, respectively; *P* = 0.002) were significantly correlated with poor neurological development (poor outcome group vs. good outcome group) (Table 2). Other neonatal factors such as birth weight SD, Apgar score, and umbilical cord blood pH, rates of CLD, ROP, circulation problem, postnatal infection, breastfeeding at discharge, and visual and hearing impairment were not related to a poor neurological outcome. The rates of IVH grade 3 (0 vs. 0.7%, respectively; *P* = 0.672) were not different between the poor outcome group and the good outcome group. In the current study, there were no cases of IVH grade 4.

**Table 2:** Neonatal factors according to neurological development.

	**Good (*n* = 140)**	**Poor (*n* = 25)**	***P***
BW (g)	1,394 (456–2,370)	1,017 (426–1,591)	**0.001**
BW SD	−0.2 (−3.6–2.6)	−1.1 (−3.5–1.5)	0.083
SGA ≤ −1.5 SD (*n*/%)	25 (17.9)	9 (36.0)	**0.039**
HC at birth (cm)	28.0 (20.3–42.5)	26.0 (19.0–28.5)	**0.002**
HC SD at birth (cm)	0 (−2–2.8)	−0.3 (−1.7–1.4)	0.275
Apgar score at 1 min	6 (1–9)	5 (1–9)	0.987
Apgar score at 5 min	8 (2–9)	8 (5–10)	0.987
UA pH	7.33 (6.93–7.60)	7.30 (7.16–7.46)	0.40
PVL (*n*/%)	3 (2.1)	4 (16.0)	**0.011**
IVH grade 3 (*n*/%)	1 (0.7)	0 (0)	0.672
RDS (*n*/%)	80 (57.1)	20 (80.0)	**0.031**
CLD (*n*/%)	51 (36.4)	14 (56.0)	0.065
NEC (*n*/%)	2 (1.4%)	3 (12.0)	**0.005**
ROP (*n*/%)	19 (13.6)	4 (16.0)	0.745
Mechanical ventilation (*n*/%)	88 (62.9)	21 (84.0)	**0.034**
NPPV (*n*/%)	114 (81.4)	23 (92.0)	0.195
Duration of oxygen supplementation (days)	35 (0–730)	71 (0–1,166)	**0.020**
Circulatory problem (*n*/%)	8 (5.7)	4 (16)	0.068
Postnatal infection (*n*/%)	7 (5.0)	3 (12.0)	0.177
Duration of NICU stay (days)	67 (22–304)	92 (29–347)	**0.002**
Breastfeeding at discharge (*n*/%)	132 (94.3)	21 (84.0)	0.068
Visual impairment (*n*/%)	13 (9.3)	2 (8.0)	0.837
Hearing impairment (*n*/%)	3 (2.1)	0 (0.0)	0.460

*BW, birth weight; SD, standard deviation; SGA, small for gestational age; HC, head circumference; UA, umbilical artery; PVL, periventricular leukomalacia; IVH, intraventricular hemorrhage; RDS, respiratory distress syndrome; CLD, chronic lung disease; NEC, necrotizing enterocolitis; ROP, retinopathy of prematurity; NPPV, non-invasive positive pressure ventilation; NICU, neonatal intensive care unit. The bold values means P < 0.05*.

Table 3 shows risk factors for a poor neurological outcome. PE complicated with maternal renal dysfunction was an independent risk factor for a poor neurological outcome (OR: 6.7, 95% CI: 1.1–40.9, *P* = 0.045). Delivery at <30 weeks of gestation was also an independent risk factor for a poor neurological outcome (OR: 3.5, 95% CI: 1.3–9.1, *P* = 0.007).

**Table 3:** Risk factors for poor neurological outcome (multiple regression analysis: *n* = 165).

	**Odds ratio**	**95% CI**	***P***
PE with maternal renal dysfunction	**6.7**	**1.1–40.9**	**0.045**
Delivery week <30 weeks′ gestation	**3.5**	**1.3–9.1**	**0.007**
SGA ≤ −1.5 SD	2.4	0.9–6.5	0.075

*CI, confidence interval; SGA, small for gestational age. The bold values means P < 0.05*.

## Discussion

To study risk factors of a poor neurodevelopmental outcome in preterm singleton children delivered at <34 weeks of gestation, we investigated 92 preterm children at a corrected age of 3 years old using Bayley-III (*n* = 91) and Kyoto Scale of Psychological Development (*n* = 1). We included follow-up children before 3 years old (*n* = 73) in the good outcome group. The rate of a poor neurological outcome was 15.2% (25/165). A total of 80% (20/25) Bayley-III scores under 85 points. Also, 80% (16/20) of those with a poor neurodevelopmental outcome had shown mild neurodevelopmental delay with Bayley-III scores of 70–84 points, and 20% (4/20) showed <70 points. Therefore, this study included those with relatively mild developmental delay.

In addition, as in previous studies, our study showed that the delivery week (28 vs. 30 weeks, respectively; *P* = 0.012), a low birth weight (1,017 vs. 1,394 g, respectively; *P* = 0.001), and SGA (36 vs. 17.9%, respectively; *P* = 0.039) were associated with a poor neurodevelopmental outcome in preterm children. However, there was no association between the onset of PE alone and a poor neurodevelopmental outcome (8.6 vs. 20%, respectively; *P* = 0.143), and it was significantly correlated with PE complicated with maternal renal dysfunction (12 vs. 2.1%, respectively; *P* = 0.045).

Also, there was no difference between IVH grade 3, low Apgar score, and poor neurological development. These results differ from those of previous reports (25–27). There were only one IVH grade 3 case and small population in our study, so a false negative could be possible. Duration of oxygen supplementation and duration of NICU stay, rates of RDS, NEC, and mechanical ventilation were significantly higher in the poor outcome group than the good outcome group. These results were consistent with those of previous reports (8, 28, 29) and considered to be strongly associated with early delivery weeks of gestation and low birth weight.

Table 3 shows that delivery at <30 weeks of gestation and PE complicated with maternal renal dysfunction were independent risk factors for poor neurological development. However, the association between the delivery week and a poor neurological outcome was previously reported (1, 2). The most meaningful and new detection of our study is that PE complicated with maternal renal dysfunction was associated with poor neurological development in preterm birth infants.

Table 4 shows the findings related to neurological impairment assessed in 1.5–11.5-year-olds, antecedently reported in offspring born to mothers with hypertensive disorder in pregnancy (HDP). Offspring whose mothers develop PE (PE-F1s) exhibit decreased cognitive function. PE was identified as a risk factor and has been associated with cognitive impairment in some studies (Nos. 1, 2, 4–14, and 16) (30, 31, 33–42, 44, 45).

**Table 4:** Findings related to neurological impairment previously reported in offspring born to mothers with hypertensive disorder in pregnancy.

**No**.	**References**	**Offspring age**	**Developmental test**	**Study design**	**Sample size**	**Delivery weeks, Birth weight**	**Findings in offspring**	**Neurological impact**
1	Szymonowicz and Yu (30)	2 years	Bayley	Case-control	35 PE vs. 35 non-PE	<32 weeks, VLBW	Lower mean MDI	Cognitive
2	Spinillo et al. (31)	2 years	Unknown	Case-control	68 PE vs. 184 non-PE	29–35 weeks	Lower MDI	Cognitive
3	Gray et al., (32)	2 years	NSMDA GQ	Cohort Follow-up	96 PIH vs. 101 NT	24–32 weeks	No cognitive impairments found	None
4	McCowan et al. (33)	1.5 years	Bayley II	Cohort Follow-up	88 PIH vs. 131 NT	Mean 36.5 weeks SGA (BW <10th centile)	Lower MDI	Cognitive
5	Many et al. (34)	3 years	Stanford Binnet-IQ	Cohort Follow-up	11 PE vs. 64 non-PE	SGA (<5th percentile) PE: 34.7 weeks, non-PE: 37 weeks	Lower IQ scores	Cognitive
6	Cheng et al. (35) (Taiwan)	2 years	Bayley	Follow-up	28 PE vs. 61 NT	<32 weeks	Lower MDI	Cognitive
7	Kronenberg et al. (36)	3.5–7 years	PDMS or MSCA	Cohort Follow-up	19 HT-AGA vs. 26 non-HT-IUGR	19 HT-AGA [mean 34.2 weeks (29–39 weeks)]26 non-HT-IUGR [mean 32.9 weeks (27–38 weeks)]	Lower cognitive and motor skills in IUGR	Cognitive
8	Spinillo et al. (37)	2 years	Bayley	Cohort Follow-up	185 PE vs. 569 NT	24-33 weeks	Lower MDI	Cognitive
9	Schlapbach et al. (38)	2 years	Bayley II	Case-control	33 PE vs. 33 non-PE	Mean 29 weeks (25–32 weeks)	Lower MDI	Cognitive
10	Van an Wassenae et al. (39)	4.5 years	Bayley	Prospective follow-up	216 children, born after severe early-onset HDP	24–34 weeks	Lower IQ scores	Cognitive
11	Whitehouse et al. (40)	10 years	PPVT-R RCPM	Cohort Follow-up	279 GH vs. 34 PE vs. 1076 NT	GH [mean 39.1 weeks (1.9 SD)], PE [mean 36.3 weeks (3.5 SD)], NT [mean 39.3 weeks (2.04 SD)]	Lower PPVT-R scores No effect of RCPM scores	Cognitive, language
12	Heikura et al. (41)	11.5 years	Wisc-R	Cohort Follow-up	198 children had mild cognitive limitations	Unknown	Mild cognitive limitation in GH	Cognitive
13	Morsing et al. (42)	5–8 years	Wechsler scales	Case-control prospective study	34 IUGR (11 PE) vs. 34 AGA (NT)	Mean 184 days (IUGR with PE), 192 days (IUGR without PE), 190 days (AGA)	Lower mean verbal IQ	Cognitive
14	Wade and Jenkins (43)	1.5 years, 3 years, 4.5 years	Unknown	Cohort Follow-up	23 HT vs. 478 non-HT	Unknown	Lower social cognition and executive function	Cognitive
15	Warshafsky et al. (44)	3 years	ASQ	Prospective cohort	129 PE vs. 140 controls	PE: 36 weeks (32–38 weeks), control 39.5 weeks (38–41 weeks)	Lower ASQ	Motor, communication, social
16	Rätsep et al. (45)	7–10 years	NEPSY-II WMTB-C WRMT WJ III Tests of AchievementSaccadic eye movement recordings	Cohort Follow-up	10 PE vs. 80 controls	Unknown	Lower working memory Lower oculomotor control	Cognitive

*PE, preeclampsia; VLBW, very low birth weight; MDI, Mental Developmental Index; NSMDA, Neuro-Sensory Motor Developmental Assessment; GQ, Griffiths' infant ability scale; PIH, pregnancy-induced hypertension; NT, normotension; SGA, small for gestational age; BW, birth weight; IQ, intelligence quotient; PDMS, Peabody Developmental Motor Scale; MSCA, McCarthy Scales of Children's Abilities; HT, hypertension; AGA, appropriate for gestational age; IUGR, intrauterine growth restriction; HDP, hypertensive disorders of pregnancy; PPVT-R, Wisc-R, Peabody Picture Vocabulary Test-Revised; RCPM, Ravens Colored Progressive Matrices; Wechsler Intelligence Scale for Children-Revised; ASQ, Ages and Stages Questionnaire; NEPSY-II, Neuropsychological Assessment, 2nd edition; WMTB-C, Working Memory Test Battery for Children; WRMT, Woodcock Reading Mastery Test-Revised; Woodcock–Johnson III Tests of Achievement*.

In our study, cognitive and language functions decreased in one case. Cognitive, language, and motor functions decreased in one case. Only language function decreased in nine cases, and only motor function decreased in four cases (Table 5). In our study, there were only two cases of cognitive decline, with the highest rate of decrease in language function. In cases of a poor neurological outcome complicated with maternal renal dysfunction (*n* = 3), one case showed a decrease only in the language function by Bayley-III, one case showed decreased language and motor functions by Bayley-III, and one case showed decreases in all areas by Kyoto Scale of Psychological Development.

**Table 5:** Areas and points in cases with <85 points by Bayley-III.

**Area**	**Points mean (min.–max.)**
Language only (*n* = 9)	81 (68–83)
Motor only (*n* = 4)	77.5 (73–79)
Language and Motor (*n* = 5)	Language 77 (47–83) Motor 76 (58–79)
Cognitive and Language (*n* = 1)	Cognitive 80, Language 74
All (*n* = 1)	Cognitive 60, Language 59, Motor 61

Three published reports (Nos. 5, 14, and 15) involved patients at the same age of 3 years old as in our study; two (Nos. 5 and 14) involved a cognitive decline; and one (No. 15) involved a motor, communication, or social decrease (34, 43, 44). Other reports examined a wide age range, from 1.5 to 11.5 years old, and the neurodevelopment test methods differ, making it difficult to compare with our study.

Figueiró-Filho et al. (46) reviewed 27 out of 464 studies reporting on the neurological development of offspring born to HDP mothers. The current studies support the behavioral and cognitive deviations in offspring whose mothers developed PE. Figueiró-Filho et al. (46) demonstrated the relation between mechanisms of PE and physiology of placenta and neurological development. From first to third trimesters of pregnancy, PE causes disorders along with cardiovascular, immune, metabolic, etc. and dysfunction of placenta such as placentation, immune tolerance, and then lead to neurological developmental disorders. PE may lead to breakdown such as hypoxia, inflammation, etc. and influence physiology and neurological development. Some knockout models of angiogenesis such as placental growth factor (PlGF) show dysfunction of angiogenesis. These knockout models indicate neurodevelopmental disorders of children of PE mothers.

The most meaningful and new detection of our study is that PE complicated with maternal renal dysfunction is related to poor neurological development in children at 3 years.

However, there has been no study on infants' neurological development focused on PE with maternal renal dysfunction. PE is related to renal dysfunction owing to deficiency in podocyte-specific VEGF (47). Placental hypoxic condition in PE cases causes elevated levels of fms-like tyrosine kinase 1 (sFlt-1) and a soluble receptor of VEGF. An increased level of sFlt-1 binds to VEGF and PlGF, which are important angiogenesis and vasculogenesis factors, resulting in endothelial dysfunction in various organs, including the kidney (48). A recent study demonstrated the effects of PlGF deficiency on the behavior, neuroanatomy, and cerebrovasculature of mice (49).

The specific character of sFlt-1 in adapting neuroanatomical and vascular construction and circulatory characteristics at the central nervous system level are unclear (50). sFlt-1 high levels are related to adult psychiatric and neurological alterations (51, 52). Vogtmann et al. (53) demonstrated that human sFlt-1 arrests placental differentiation, exclusively arresting fetal capillary branching by decreasing VEGF signaling and inducing apoptosis in fetal embryonic stem cells. This arrest could lead to a stagnation of maternal blood, improving dilatation of the maternal sinusoids according to damage of the fetal vessel system. The changed morphology of placenta finally results in insufficiency of utero and placenta, containing decreased nutrient transport (chiefly influencing the fatty acid supply) and induction of FGR (53).

High sFlt-1 levels are considered to cause glomerular damage, proteinuria, and renal damage. In cases of PE complicated with renal dysfunction, hyperproteinemia can lead to malnutrition of the infant and neurological adverse effects as a result of systemic vascular endothelial dysfunction.

Barker et al. (13) showed the differentiation of risk factors for adult diseases after adulthood depending on the stage of pregnancy when the mother was exposed to starvation and those born to mothers who are underweight at birth are at higher risk of developing cardiovascular disease, diabetes, and hypertension. A mechanism exists in the fetus to adapt (e.g., expression of a thrifty gene) to environmental stimuli (insufficient supply of nutrients from the placenta to the fetus due to malnutrition of the mother) in each organ and tissue, and this program also exists in adulthood. It is hypothesized that it increases the risk of developing the disease (54, 55).

Fetal growth in the uterus is by cell division and proliferation in all tissues, and undernutrition during pregnancy leads to fetal organs and placenta showing low cell numbers (56). From this experiment, fetal undernutrition, low protein nutrition, and early postnatal malnutrition may cause brain damage.

Offspring of mothers with PE complicated with maternal renal dysfunction, hyperproteinemia, and undernutrition may show a poor neurodevelopmental outcome as preterm infants.

## Strengths and Limitations

This study is the first report to show the relationship between PE with maternal renal dysfunction and infants' poor neurological development.

The strengths of the present study include neurological development being evaluated by Bayley-III in Japan. In Japan, there have been only seven studies about neurodevelopment of infants assessed by Bayley-II or III (57–63). However, there has been only one study on preterm infants among these reports (63). A previous study compared 23 low-birth weight infants diagnosed with PVL (median gestational age: 30 weeks) with 209 normal-weight infants (gestational age range: 25–39 weeks). In this study, we reported the neurological development of Japanese preterm infants evaluated by Bayley Scales of Infants and Toddler Development for the first time.

Major limitations of our study are retrospective study design and small sample size in a single institution. Also, this study did not involve cases of severe developmental delay, only relatively mild developmental delay. Only 91 cases (55.2%) out of 165 included infants were evaluated with the Bayley-III method as the selected tool to assess the neurodevelopmental outcome. There is attrition bias. Ideally, all cases should have Bayley-III assessment at 3 years old. However, rest of all cases (73 cases, 44.2% out of 165 cases) were followed up until the neonatologists diagnose they are normally developed and instructed to see a neonatologist/pediatrician in our hospital if they had developmental problems at health checkup at 3 years old. Our data did not include toxic exposure such as NICU noise and social deprivation such as poor socioeconomic status, so there is a relevant bias. Bayley-III was originally developed by American norms, so there could be a difference between Japan norms and American norms.

## Conclusion

Offspring of mothers with PE complicated with maternal renal dysfunction required long-term neurodevelopmental follow-up.

## Data Availability Statement

The raw data supporting the conclusions of this article will be made available by the authors, without undue reservation.

## Ethics Statement

The studies involving human participants were reviewed and approved by The Institutional Review Board of Toyama University Hospital. Written informed consent to participate in this study was provided by the participants' legal guardian/next of kin.

## Author Contributions

NY: conception of the work, analysis, interpretation, and writing this paper. SY, ST, MI, AS, HN, TY, and AN: contribution to the design of the work, revising it critically for important intellectual content, agreement to be acccountable for all aspects of the work. All authors contributed to the article and approved the submitted version.

## Conflict of Interest

The authors declare that the research was conducted in the absence of any commercial or financial relationships that could be construed as a potential conflict of interest.
